# Effects of exercise training in 23 adults with Pompe disease receiving enzyme therapy

**DOI:** 10.1186/1471-2474-14-S2-P17

**Published:** 2013-05-29

**Authors:** Linda van den Berg, Marein Favejee, Stephan Wens, Michelle Kruijshaar, Francois Karstens, Robert Rozenberg, Stephan Praet, Arnold Reuser, Pim Pijnappel, Johannes Bussmann, Pieter van Doorn, Harm Kuipers, Ans van der Ploeg

**Affiliations:** 1 Center for Lysosomal and Metabolic Diseases, Erasmus MC University Medical Center, Rotterdam, The Netherlands; 2 Department of Rehabilitation Medicine & Physical Therapy, Erasmus MC University Medical Center, Rotterdam, The Netherlands; 3 Department of Movement Sciences, School for Nutrition, Toxicology and Metabolism, Maastricht University, Maastricht, The Netherlands

## Introduction

Pompe disease is a metabolic myopathy caused by the deficiency of acid α-glucosidase. In addition to enzyme replacement therapy (ERT), exercise training may help improve patients’ fitness and physical functioning. Thus, we studied the safety and efficacy of exercise training in adult Pompe patients.

## Methods

Inclusion criteria were:

1. ERT ≥ 1 year,

2. no walking aids,

3. no ventilators.

The 12-week training program consisted of aerobic, resistance, and core stability exercises in 36 sessions. Plasma creatine kinase (CK) was measured biweekly. Aerobic fitness, muscle strength, muscle function, core stability and body composition were evaluated before and after the program.

## Results/discussion

23 patients successfully completed the training program with no significant side-effects. Aerobic fitness improved, shown by increases in workload (100W ± 52 to 122W ± 53, p<0.01), maximal oxygen uptake (69.4% of normal ± 17.4 to 75.9% of normal ± 18.0, p<0.01), and anaerobic threshold (16.7 ± 4.3 ml/min/kg to 18.5 ± 4.7 ml/min/kg, p=0.01). Small increases were observed in total muscle strength and in proximal lower extremities using hand-held dynamometry (both p=0.01). This increase was mainly due to an eight percent-point increase in strength of the hip flexors (p<0.01). At the end of the program, timed muscle function tests indicated that patients took significantly less time to climb four stairs (0.3 second less, p=0.04) and rise from supine to standing (1 second less, p=0.03). The time to run 10 meters did not significantly change. The number of patients able to perform the core stability exercises rose during the training program, and the total time patients were able to remain in balance improved dramatically for all four positions (58% increase for the backbridge, 229% and 223% for the left and right side bridges, respectively, and 86% for the abdominal bridge; p<0.05). No changes in body composition were found.

## Conclusion

This is the first study to prove that a combination of aerobic, strength and core stability training can be safely performed and leads to improvements in aerobic fitness, muscle strength, muscle function, and core stability in patients with Pompe disease receiving long-term ERT.

